# Harnessing the Power of Invariant Natural Killer T Cells in Cancer Immunotherapy

**DOI:** 10.3389/fimmu.2017.01829

**Published:** 2017-12-18

**Authors:** Melissa Bedard, Mariolina Salio, Vincenzo Cerundolo

**Affiliations:** ^1^MRC Human Immunology Unit, Weatherall Institute of Molecular Medicine, University of Oxford, Oxford, United Kingdom

**Keywords:** invariant natural killer T cells, CD1d molecules, tumor immunology, innate immune response, lipid antigens

## Abstract

Invariant natural killer T (iNKT) cells are a distinct subset of innate-like lymphocytes bearing an invariant T-cell receptor, through which they recognize lipid antigens presented by monomorphic CD1d molecules. Upon activation, iNKT cells are capable of not only having a direct effector function but also transactivating NK cells, maturing dendritic cells, and activating B cells, through secretion of several cytokines and cognate TCR-CD1d interaction. Endowed with the ability to orchestrate an all-encompassing immune response, iNKT cells are critical in shaping immune responses against pathogens and cancer cells. In this review, we examine the critical role of iNKT cells in antitumor responses from two perspectives: (i) how iNKT cells potentiate antitumor immunity and (ii) how CD1d^+^ tumor cells may modulate their own expression of CD1d molecules. We further explore hypotheses to explain iNKT cell activation in the context of cancer and how the antitumor effects of iNKT cells can be exploited in different forms of cancer immunotherapy, including their role in the development of cancer vaccines.

## Introduction

The evidence that peptide-specific T cells play an important role in the immune defense against pathogens and cancer progression is compelling (1–3). In the last two decades, it has emerged that in addition to peptide-specific T cells, hereafter referred to as conventional T cells, α/β T cells can also recognize lipids and metabolites of vitamin B2 in the context of monomorphic MHC class I-like molecules (4, 5). Such T cells, hereafter referred to as unconventional T cells, can orchestrate an immune response against pathogens and cancer (4). This review will focus on the description of one of these unconventional T cell populations—invariant natural killer T (iNKT) cells. We will also highlight potential mechanisms of iNKT cell activation in cancer and how these cells can be manipulated for the purpose of cancer immunotherapy.

### Development and Function

Invariant natural killer T cells originate from bone marrow-derived progenitors that, like conventional T cells, migrate to the thymus. However, unlike conventional T cells, which are selected by self-peptides presented by MHC class I and II on thymic epithelial cells, iNKT cells are positively selected by CD1d molecules expressed by double-positive (CD4^+^CD8^+^) thymocytes (6, 7). Such CD1d molecules present self-lipid ligand(s), not yet fully characterized, and upon expression of the transcription factor PLZF, the thymocytes acquire the iNKT cell effector program (8). iNKT cells subsequently migrate out of the thymus and reach maturity in the periphery (8). Unlike naïve conventional T cells, iNKT cell numbers in humans are high, particularly in the spleen and in the liver, reaching about 1% of total lymphocytes in the latter tissue (9).

Invariant natural killer T cells are considered innate-like lymphocytes as they exhibit characteristics of both innate and adaptive immune cells. Their activation is driven by antigen recognition, a characteristic of conventional adaptive immune cells. However, unlike conventional T cells, iNKT cells bear a semi-invariant TCR that recognizes different lipid antigens presented on monomorphic CD1d molecules. This recognition manner has been likened to a pattern recognition mode (10). iNKT cells further deviate from conventional T cells by their ability to rapidly secrete copious amounts of cytokines, mainly IFN-γ and IL-4, shortly upon activation—a characteristic reminiscent of innate immune responses, and which is imparted at the epigenetic level by their unique developmental program (11–14).

Since iNKT cells are uniquely placed at the interface between innate and adaptive immunity, they have a tremendous influence in shaping immune responses. Cytokine stimulation and cognate interaction between iNKT cells and dendritic cells (DCs), B cells, neutrophils, and macrophages often polarizes these cells toward a pro-inflammatory phenotype (15–25). Similarly, activated iNKT cells can transactivate natural killer cells (26) and enhance stimulation of conventional T cells through their ability to secrete cytokines and mature DCs (16, 18). Although the frequency of iNKT cells in humans ranges from 0.01 to 0.1% in peripheral blood (lower than in mice), this frequency is still orders of magnitude higher than that of naïve peptide-specific T cells (9, 27). In addition, their constitutive expression of CD40L and ability to rapidly secrete cytokines make iNKT cells critical players in immunity, by orchestrating all-encompassing immune responses (9).

### Means of Activation

There are two primary means of iNKT cell activation: CD1d-dependent and cytokine-driven activation. CD1d molecules are transmembrane proteins that, similar to MHC class I molecules, bind non-covalently to β_2_-microglobulin. The surface-exposed antigen-binding groove consists of two deep hydrophobic channels that bind the fatty acid tails of lipid antigen, while the head moiety is exposed for recognition by the iNKT-TCR (28, 29). Ceramide-based glycolipids (glycosphingolipids) and glycerol-based lipids (such as membrane phospholipids) are the two main types of iNKT-activating lipids bound to CD1d molecules (30–34). While the most potent iNKT-activating lipid agonists described to date is threitol-6-ceramide (35), the classical iNKT-activating lipid agonist most frequently used in the literature is α-galactosylceramide (αGC), which is derived from a bacterium on the *Agelas mauritianus* marine sponge (23, 36–38). Analysis of the crystal structure of CD1d monomers with or without αGC, which exploits the full binding capacity of CD1d, allowed for the identification of the hydrogen bonds required to hold the polar head of iNKT cell agonists (29). The presence of both a lipid binding and non-lipid binding molecule in the asymmetric unit of the CD1d crystals has enabled the identification of two different conformations of the antigen-binding groove (29). Using planar lipid bilayers and surface plasmon resonance, the contribution of the length and saturation of the alkyl chains occupying the A′ and F′ channel of human CD1d molecules to the stability of CD1d-lipid complexes and to the affinity of iNKT-TCR binding was further analyzed (39). These results led to the description of a general mechanism by which the length of the lipid chain occupying the F′ channel plays a role in controlling the affinity of lipid-specific CD1d-restricted T cells (39). This concept can be more generally extend to other CD1-restricted cells (40).

In a more physiological context, iNKT cells become activated by microbial or self-lipid antigens bound to CD1d molecules. For example, isoglobotriosylceramide (iGB3), a neutral glycosphingolipid, has been identified as a weak self-lipid antigen for human and murine iNKT cells (41–43), although its role as the only positive-selecting self-lipid in the thymus remains controversial, given that mice lacking the required synthases for iGB3 production maintain an intact iNKT cell repertoire (44, 45). Lysophospholipids and charged glycosphingolipids have been shown to be self-lipid antigens in different contexts (46–48). Self-lipid antigens are weakly immunogenic and iNKT cell activation in this case is often largely driven by IL-12 and IL-18. In a model of hepatitis B infection, it has been shown that viral-induced phospholipases generate lysophospholipids that lead to iNKT cell activation (30, 47).

Cytokine-driven activation is common when lipid antigen is weakly immunogenic (47). Although CD1d-activated iNKT cells can undergo further activation *via* cytokines secreted from matured DCs, certain cytokines, namely IL-12 and IL-18, are alone sufficient to activate iNKT cells (49, 50). Avidity might play a more important role in iNKT cell activation than previously considered, especially iNKT cell activation by self-lipid antigen repertoire. Alterations in the actin cytoskeleton are evidenced to create CD1d nanoclusters of higher avidity, increasing basal iNKT autoreactivity (51).

## iNKT Cells in Antitumor Immunity

The ability of iNKT cells to orchestrate immune responses against cancer is perhaps the most striking example of their role in disease. Work from the laboratory led by Dale Godfrey highlighted the essential role of iNKT cells in tumor immunity by demonstrating that mice lacking iNKT cells were more susceptible to methylcholanthrene-induced sarcomas, consistent with the role of iNKT cells in immunosurveillance (52). This effect was reversed upon iNKT cell reconstitution, an observation that further supports their role in tumor clearance. Although the antitumor effector activity of iNKT cells upon αGC injection was recently confirmed using newly generated Jα18-deficient mice, which bear an otherwise normal T cell repertoire (53), the role of iNKT cells in immunosurveillance of methylcholanthrene-induced sarcomas was called into question in a separate study (54).

Invariant natural killer T cells’ ability to modulate various immune subsets is key to their role in antitumor immune responses. iNKT cells can mature DCs, activate CD4^+^ T cells, CD8^+^ T cells, and B cells, and transactivate NK cells (19, 23). In murine models of lung and liver cancers, the antitumor effect of αGC administration was attributed to IFN-γ secretion from iNKT cells and transactivated NK cells, which culminated in NK perforin-mediated cytolysis of tumor cells (23). iNKT cell-derived IFN-γ is also responsible for enhanced activation of tumor antigen-specific CD8^+^ T cells (19, 55, 56). Additionally IL-12 derived from iNKT cell-matured DCs helped priming of tumor antigen-specific T cells (19, 57).

Invariant natural killer T cells can also augment an antitumor response by diminishing the immunosuppressive activities of immune subsets that promote tumorigenesis. It has been shown that iNKT cells can have a profound effect on the number and function of pro-tumorigenic myeloid populations (22, 58, 59). Tumor-associated macrophages (TAMs), which secrete immunosuppressive molecules such as IL-6 and TGF-β that dampen T-cell responses to MHC-presented tumor antigen, are found in the tumor microenvironment of a variety of cancers, including renal cell carcinomas and neuroblastoma (59). In primary human neuroblastoma samples, iNKT cells specifically killed the tumor-antigen-loaded TAMs rather than neuroblastoma cells, in part relieving the immunosuppressive tumor microenvironment and limiting metastases (59). iNKT cells are also capable of reducing myeloid-derived suppressor cells (MDSC) numbers and immunosuppressive activity (22, 58). These findings beg the question of how iNKT cells remain unaffected by the immunosuppressive microenvironment. It is reported that in patients with head and neck cancer, iNKT cells, unlike conventional T cells, are resistant to hydrogen peroxide produced by CD15^+^ MDSCs (60). This observation potentially explains their persistent activation and cytotoxic activity within an immunosuppressive tumor microenvironment.

While iNKT cells are best known to potentiate their antitumor effect through enhancing the immunogenic activities of a variety of immune cell subsets, they are capable of themselves recognizing and killing CD1d^+^ tumor cells. Such is true for the EL4 T-cell lymphoma model, where both *in vitro* and *in vivo* iNKT cells directly executed perforin-mediated cytolysis of lymphoma cells in a CD1d-dependent manner (61, 62). Furthermore, in a TRAMP murine model of CD1d^+^ prostate cancer, iNKT cells directly and predominantly reduce tumorigenesis, to a greater extent than cytotoxic T lymphocytes (63). In addition, in naturally expressing CD1d^+^ human osteosarcoma cell lines, iNKT cells selectively killed the tumor cells through Fas-FasL interaction, while leaving cocultured CD1d^−^ osteoclasts and CD1d^+^ mesenchymal stem cells unaffected (64). Glioma and breast cancer cell lines transduced with CD1d are targets of iNKT cell-dependent lysis (65, 66). These results collectively indicate that iNKT cells are capable of directly killing CD1d^+^ tumors. In the large proportion of cases where solid tumors are CD1d^−^, tumor-infiltrating CD1d^+^ myeloid populations might activate iNKT cells either within the tumor or in distal lymphoid tissues enriched in iNKT cells.

Though their activation can contribute greatly to antitumor immune responses, there is a dearth of evidence that iNKT cells are present within the tumor microenvironment, particularly in human solid tumors. This issue might stem from low frequency of iNKT cells in humans, potentially making them difficult to detect by immunohistochemical techniques. While iNKT cell-specific antibodies, including the 6B11 antibody (67), do exist, there are few reports of their use in identifying iNKT cell populations within tumor microenvironments.

## Modulation of CD1d: Tumor Evasion from iNKT Cell Immunosurveillance

**Figure 1:**
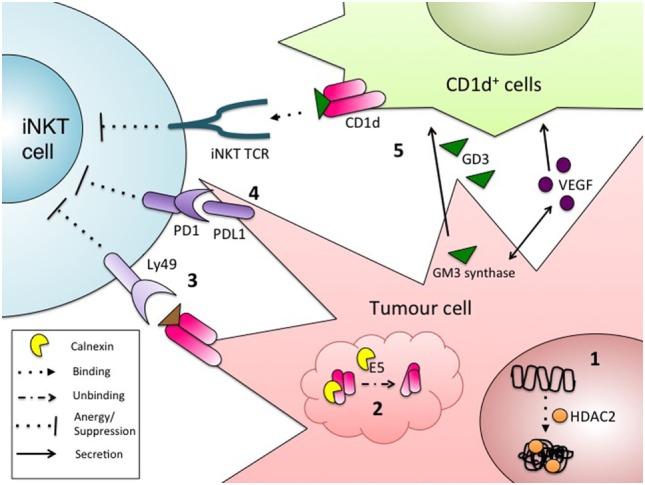
Mechanisms of tumor evasion from invariant natural killer T (iNKT) immunosurveillance. Some tumors cells escape detection by iNKT cells *via* the regulation of surface CD1d, by: **(1)** heterochromatin formation at the CD1d locus by histone deacetylases (71, 72); or **(2)** improper folding and retention of CD1d in the ER (69). Other mechanisms to escape iNKT cell detection include: **(3)** engagement of surface CD1d with the inhibitory NK receptor Ly49, leading to the induction of iNKT cell anergy (70); **(4)** inhibitory signaling through PD1/PDL1 between iNKT cells and tumor cells (139); and **(5)** CD1d-dependent suppression of iNKT cells through presentation of the inhibitory, tumor-derived glycolipid GD3. GD3 production is also driven by the secretion of tumor-derived VEGF (75).

Tumor cells use a variety of mechanisms to escape detection and elimination by immune cells. These mechanisms include releasing soluble mediators to dampen antitumor immune responses, notably TGF-β into the microenvironment and inducing T cell anergy and exhaustion (68). Another mechanism involves hindering antigen presentation, often by limiting expression of antigen-presenting molecules in infiltrating myeloid cells or on the tumor cells themselves. MHC class I molecules, which present peptide antigens to CD8^+^ T cells, are well characterized as a target of such escape mechanisms (68). Similarly, a variety of tumors downregulate CD1d molecules, further emphasizing the important role of iNKT cells in antitumor immunity (Figure 1).

The correlation between reduced CD1d expression and enhanced tumor progression has been reported in a variety of types of CD1d-transduced solid cancers, including breast, cervical, ovarian, prostate, lung, and melanoma (66, 69–72). This observation holds true for many naturally CD1d^+^ and transduced CD1d^+^ liquid tumors, such as mantle cell lymphoma, multiple myeloma, and chronic lymphocytic leukemia (61, 71, 73, 74). However, different tumors engage different mechanisms to reduce CD1d surface expression. On the RNA level, modulation of CD1d expression is largely driven by epigenetic changes. Treatment of mantle cell lymphoma cell lines with histone deacetylase inhibitors resulted in enhanced iNKT cell activation upon coculture (71). This observation was attributed to the removal of HDAC2 from the CD1d promoter, resulting in increased CD1d expression (71). Another report, which substantiates these findings, demonstrated that treating human and murine lung cancer and melanoma cell lines with HDAC2 inhibitors induce CD1d expression, although the functional relevance was not investigated (72). CD1d assembly in the endoplasmic reticulum (ER) is another potential target for tumor CD1d downregulation. In a model of HPV-driven cervical cancer, early-infected epithelial cells exhibited reduced CD1d expression compared to uninfected cells. In infected cells, the viral protein E5 inhibited calnexin, resulting in improper folding of CD1d, retention of CD1d molecules in the ER, and subsequent proteasomal degradation (69).

Modulation of iNKT cell function, even when CD1d molecules reach the surface of tumor cells, can contribute to evasion of iNKT surveillance. In the TRAMP murine model of prostate cancer, tumor cells express functional CD1d molecules, but lead to aberrant iNKT-cell activation akin to anergy, likely through the inhibitory receptor Ly49 (70). This phenotype could be rescued by simultaneous stimulation with αGC and IL-12, which likely overrides the inhibitory signal.

Tumor-derived factors can also inhibit trans-CD1d-dependent antigen presentation. When murine CD1d^+^ fibroblasts were treated with human ascites from ovarian cancer patients, CD1d-dependent iNKT cell activation was markedly reduced, suggesting that a soluble factor released from ovarian tumors could affect CD1d-dependent activation (75). VEGF, a pro-angiogenic and pro-tumorigenic soluble factor, and the suppressive glycolipid antigen GD3, were identified as the factors present in the ascitic fluid inhibiting iNKT cell activation (75). Interestingly, the authors also showed that GD3 synthesis was dependent on VEGF-mediated upregulation of GM3 synthase in the ovarian cancer cells (75).

While these findings illustrate the importance of CD1d in mounting iNKT-driven antitumor immune responses, there exists at least one example where increased CD1d expression and tumor progression are positively correlated (76, 77). Through microarrays, immunohistochemistry, and patient statistics, enhanced CD1d expression was associated with increased malignancy and higher relapse rates in a subset of human renal cell carcinoma, clear cell renal carcinoma (76). This result serves as a rare example of enhanced CD1d expression as a predictor of tumor progression. It is possible that CD1d-dependent activation of suppressive type II iNKT cells, to be discussed later, might contribute to this phenotype (78, 79).

Both tumor cells and tumor-infiltrating immune cells are subject to microenvironmental stress due to nutrient deprivation, hypoxia, or accumulation of toxic products of catabolism (80). This suboptimal environment can lead to upregulation of autophagy, a survival-promoting pathway centered on lysosomal-recycling intracellular material (80). Tumor cells that engage the autophagy pathway become more robust and are able to better persist and metastasize (80). It has been recently shown that in murine bone marrow DCs, deletion of the autophagy regulator protein ATG5 led to increased CD1d-dependent antigen presentation, due to limited CD1d internalization (81). However, it has also been demonstrated that during thymic iNKT cell development, ATG5 is dispensable to CD1d expression (82, 83). More research is required to clarify the role of autophagy in CD1d expression, as perhaps this mechanism is cell and time dependent.

## iNKT Cell Activation in Sterile Inflammation—Cancer

**Figure 2:**
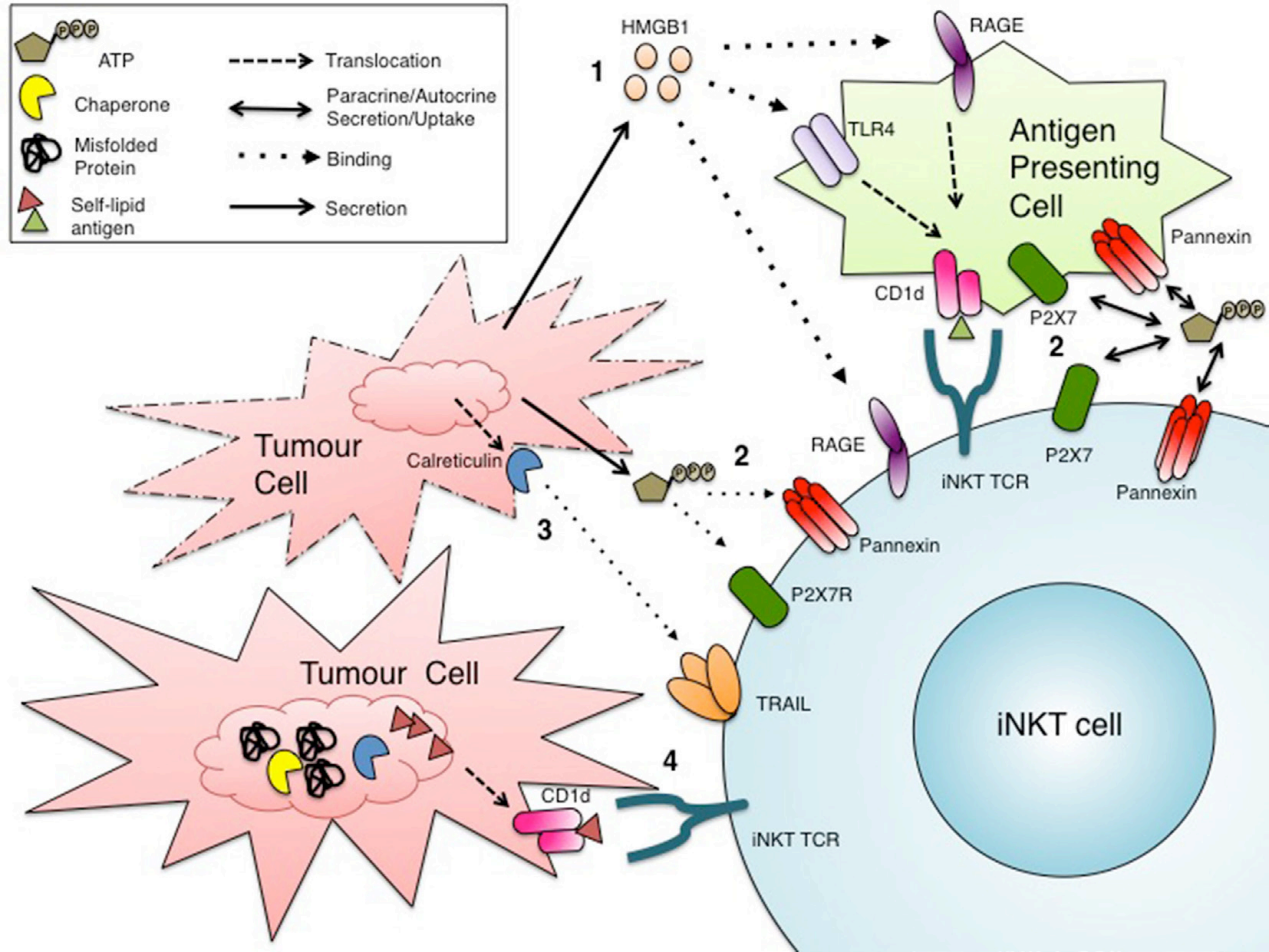
Potential mechanisms of invariant natural killer T (iNKT) cell activation in cancer. Tumor cells subject to drugs or conditions that induce stress might activate iNKT cells through several pathways: **(1)** secretion or passive release of DAMPs, such as HMGB1, that bind RAGE receptors directly on iNKT cells or TLR4/RAGE receptors on CD1d+ antigen-presenting cells (APCs), leading to the presentation of an immunogenic self-lipid antigen (46, 88, 90); **(2)** paracrine (from APCs) or autocrine release of ATP into the extracellular environment for uptake by iNKT cells, ultimately leading to iNKT cell activation (94, 95); **(3)** binding of the surface DAMP calreticulin to TRAIL on iNKT cells (103–110); **(4)** induction of ER stress in CD1d+ cells, due to the suboptimal physiological tumor microenvironment, might trigger the alternate loading immunogenic self-lipid antigen(s), resulting in enhanced iNKT cell activation (111–113).

The modulation of CD1d expression in tumor cells provides strong evidence for the critical role of iNKT cells in mounting antitumor immune responses. However, it remains unclear how iNKT cells become activated in the context of cancer, a form of sterile inflammation. The characterization of a growing number of activating stimuli and pathways, some of which might affect lipid antigen presentation, sheds light on a number of mechanisms that might contribute to iNKT sterile activation in cancer (Figure 2).

At the intersection of chemotherapy and immunotherapy lies a class of drugs that provoke a type of cell death in tumor cells that results in the activation of innate immune cells. This type of cell death is termed immunogenic cell death (ICD). When cancer cells die, for example, by necrosis, they surface-expose or release molecules called danger-associated molecular patterns (DAMPs) that are usually contained within the cell, not on the cell surface or in the extracellular milieu (84). These DAMPs can be recognized by receptors on a variety of immune cells, including toll-like receptors (TLRs), and initiate an immune response (84).

One prototypic DAMP is a protein called high mobility group box 1 (HMGB1). HMGB1, ubiquitously expressed in a variety of cell types, typically resides in the nucleus as a chromatin binding protein (85). However, HMGB1 can be released into the extracellular environment where it behaves as a DAMP (85). Its release may be passive or mediated by an active mechanism. In the passive form, HMGB1 is released from cells dying by necrosis or other forms of ICD (86). In the active form, HMGB1 is secreted from myeloid cells, namely DCs and macrophages (87). In the extracellular milieu, HMGB1 can bind and signal through TLRs 2, 4, and the receptor for advanced glycation end products (RAGE) expressed on innate immune cells, triggering an immune response (86). RAGE is reportedly expressed on iNKT cells and can bind HMGB1, resulting in a Th17 activation profile (88). While the presence of TLRs on iNKT cells is disputed, most CD1d^+^ cells also bear TLRs (46, 89–91). Viral signaling through TLR7 on human DCs has also been implicated in enhanced *de novo* synthesis of CD1d molecules (91). Furthermore, engagement of TLR4 on CD1d^+^ myeloid cells, both murine and human, enhanced loading of self-lipid antigens onto CD1d molecules leading to iNKT cell activation (46, 90). These findings give rise to the possibility that HMGB1 signaling through TLR4 could induce loading of immunogenic self-lipid antigens onto CD1d, thus providing an explanation for iNKT cell activation in cancer.

Another soluble DAMP involved in ICD in cancer is adenosine triphosphate (ATP) (92, 93). ATP can interact with various immunomodulatory receptors and channels on myeloid cells and lymphocytes, a process termed purinergic signaling/regulation (94). Myeloid cells and T cells uptake extracellular ATP through P2X7 or pannexin channels, which can enhance inflammasome activation and amplify TCR-mediated activation, respectively (94). This mode of purinergic signaling might be especially relevant in the context of cancer, where stressed and necrotic tumor cells could release ATP into the tumor microenvironment and in turn augment TCR stimulation in response to weak tumor antigens (94). Like conventional T cells, iNKT cells bear P2XR and pannexins that allow uptake of extracellular ATP, which could provide additional costimulation to CD1d-mediated activation (95). iNKT cells also express two ectonucleotidases, CD39 and CD73 (95). CD39 converts ATP, a pro-inflammatory mediator to ADP, which is in turn converted by CD73 into AMP, an anti-inflammatory mediator (96). This step-wise generation of AMP from ATP is thought to cause a shift toward an immunosuppressive microenvironment, which might be advantageous for tumor progression (96). While the balance between extracellular ATP release and catabolism in the tumor microenvironment is poorly understood, we are gaining insights into mechanisms underlying purinergic signaling in iNKT cells.

The A2 adenosine receptor (A2AR), which binds adenosine to shift immune cells toward an immunosuppressive phenotype, also has a great influence on iNKT cell activation. In a model of concanavalin A-induced hepatitis, which is predominantly iNKT cell dependent, severity of the disease phenotype was dependent on the strength of A2A receptor signaling, with an exaggerated version of the disease seen in A2AR^−/−^ mice, and an abrogation of the disease phenotype in mice treated with an A2AR agonist (97). It was later determined that A2AR exerts control over cytokine secretion in iNKT cells, particularly IL-4 and IL-10 (98).

Adenosine triphosphate is also involved in DC–iNKT cell interactions culminating in the release of inflammatory mediators that promote neutrophil recruitment. In monocyte-derived DC and iNKT cocultures, release of ATP from one or both of these immune cells (the secreting cell type was not identified) induced calcium flux within the DC through P2X7 signaling, which in turn triggered the release of prostaglandin E2 and soluble factors that promote neutrophil recruitment (99, 100). These observations were made in the setting of sterile inflammation, hinting that the influence of ATP in iNKT–DC interactions could hold true in the context of cancer (99). Maturation and stimulation of different immune cell populations by iNKT cells are thought to underlie their ability to induce antitumor immunity. This observation might be explained by the influence of purinergic signaling leading to the recruitment of neutrophils and perhaps other cell types.

Another potentially relevant soluble factor is heat shock protein 70 (Hsp70) an ER-derived chaperone (101). Similar to HMGB1, Hsp70 is upregulated during a variety of stress conditions, and under extreme stress conditions that induce tissue injury or necrotic cell death, Hsp70 is released in the extracellular environment (101). Hsp70, or more specifically the Hsp70-derived 14-amino acid peptide, in combination with either IL-2 or IL-15, enhanced the expression of NK-activating receptors, including their expression on the surface of iNKT cells (102). It remains to be seen whether this observation drives iNKT cell activation in the context of cancer.

Invariant natural killer T cells upregulate a number of different receptors upon activation, including activation markers such as CD69 and CD25, and cytotoxicity molecules including FASL and TNF-related apoptosis-inducing ligand (TRAIL) (103). TRAIL, best known for inducing apoptosis in cells expressing TRAIL receptors such as DR4 and DR5, shares a high degree of homology with FASL in the extracellular binding motif (104). Although FASL-mediated killing is often indiscriminate due to rather ubiquitous expression of FAS on mammalian tissues, TRAIL-mediated cytotoxicity is more selective toward virally infected cells and tumor cells, making it a potential target in immunotherapy (104). In humans, TRAIL is upregulated on iNKT cells upon activation and is consequently able to induce apoptosis is acute myeloid leukemia (AML) cells (105), which bear TRAIL receptors (103). This finding is substantiated in an AML murine model (103). Upon αGC administration, iNKT-derived IFN-γ upregulated TRAIL expression on activated NK cells, which in turn limited the metastasis of liver and lung tumors (106). While TRAIL has a number of well-recognized receptors, a less characterized interaction is its binding and signaling through calreticulin. Under normal circumstances, calreticulin is retained in the ER where it acts as a chaperone. However, under conditions of extreme stress leading to ICD, as is often the case with tumor cells during chemotherapy, calreticulin can be translocated to the surface of dying cells (107). In fact, calreticulin is a marker of ICD and is considered a DAMP. Soluble TRAIL has been found to interact with calreticulin expressed on A375M melanoma cells (107). Furthermore, calreticulin exposure on malignant AML blasts is correlated with increased frequency of T lymphocytes and improved survival—a finding that complements TRAIL^+^ iNKT cells’ killing of AML cells, although in this cohort of AML patients iNKT cells were not investigated (108). While the link between calreticulin-TRAIL cognate interaction and iNKT-dependent tumor killing requires further corroborating research, it would potentially provide a molecular mechanism for the iNKT cell-mediated antitumor effects in a variety of cancers.

Cells subject to TRAIL-induced killing typically undergo apoptosis, regulated non-inflammatory cell death, or necroptosis, a form of regulated inflammatory cell death due to the release of DAMPs (109). Both apoptosis and necroptosis utilize RIPK1/3 signaling (109). In TRAIL-mediated cytotoxicity, the switch between the two types of death is dictated by the acidity of microenvironment. It is tempting to speculate that in the tumor microenvironment, in which nutrients are in short supply and hypoxia is a hallmark, the consequent acidic surrounding might shift TRAIL-mediated cell death towards necroptosis. Indeed, TRAIL-induced necroptosis can contribute to *in vitro* killing of human HepG2 liver and HT29 colon cancer cell lines. iNKT cells also induce TRAIL-mediated necroptosis in a ConA model of hepatitis (110). Although iNKT cells exert cytolytic functions via necroptotic signaling in target cells, aspects of the necroptotic signaling pathway are also essential in iNKT cells themselves.

RIPK3, a kinase involved in the transduction of the necroptotic signaling pathway, regulates iNKT cell activation independent of necroptosis, as RIPK3 knockdown iNKT cells exhibited impaired cytokine secretion, including IFN-γ, upon αGC stimulation (110). Furthermore, wild-type mice inoculated with B16 melanoma were able to clear the tumor burden upon administration of αGC, but RIPK3^−/−^ mice were unable to do so, suggesting that RIPK3 is essential in iNKT-mediated anti-tumor responses (110). Further dissection of the mechanism involved illustrated that RIK3 signaling can induce the mitochondrial phosphatase PGAM5, which in turn upregulates NFAT translocation into the nucleus and stimulates the mitochondrial GTPase Drp1 (110). These factors appear to regulate TCR- and cytokine-mediated iNKT cell activation (110). These findings lay the foundation for a new pathway that can be manipulated in therapies centered on enhancing iNKT cell activation.

Cancer cells are subject to rapid cell division, leading to a reduction in available nutrients in the microenvironment and accumulation of nascent and/or mutated proteins. These suboptimal conditions, both intrinsic and extrinsic to the cell, compromise ER homeostasis and trigger the unfolded protein response (UPR) (111). Additionally, the UPR is likely triggered in immune cells in the suboptimal tumor microenvironment. UPR activation in malignant and infiltrating immune cells would alter lipid biosynthetic pathways (112). UPR activation might lead to sorting of self-lipid antigens onto CD1d complexes on CD1d^+^ tumor or surrounding immune cells—which in conjunction with inflammatory cytokines might become immunogenic. It has been shown that the microsomal triglyceride transfer protein (MTTP) lies at the intersection between the UPR and CD1d–lipid complex formation (113). MTTP forms a heterodimer with protein disulfide isomerase (PDI) and transfers different lipid antigens onto assembling CD1d complexes in the ER. Importantly, PDIs are upregulated during UPR activation (112, 113). It is thus possible that ER-stressed CD1d^+^ cells exhibit altered self-lipid loading, such that immunogenic self-lipid antigens are presented to and activate iNKT cells in the context of cancer and other forms of sterile inflammation (113).

## Suppressive NKT Cells—A Role in Promoting Tumor Progression

While iNKT cells are generally thought to augment antitumor immune responses, there exist subsets of NKT cells that exhibit a regulatory phenotype, which in fact might hinder antitumor responses and promote tumor progression. One of the earliest reports suggesting the presence of regulatory NKT cells identified that IL-13 secreted from NKT cells could signal through the IL4R-STAT6 pathways in cytotoxic T lymphocytes and consequently hinder their immunosurveillance of colon carcinoma and fibrosarcoma tumors (114). NKT cell-derived IL-13 can further drive impaired tumor immunosurveillance by inducing TGF-β secretion from a population of myeloid cells (115). These findings led to the identification of a Vα14Jα18^−^ CD1d-restricted NKT population, dubbed type II NKT cells, which regulate and suppress antitumor immunity independent of IL-4, in contrast with the better-characterized Vα14Jα18^+^ CD1d-restricted iNKT population, or type I iNKT cells, that augment antitumor immunity (79). However, in a murine model of osteosarcoma, CD1d-restricted NKT cells activated an immunoregulatory pathway independent of IL-13, IL4R-STAT6 signaling, and TGF-β, suggesting the existence of an alternative mechanism of NKT-mediated immunoregulation or different subsets of immunoregulatory NKT cells in different tumors (116). The regulatory contribution of type II NKT cells compared to classical Tregs was explored in a murine model of colorectal and renal cancers (117). This work indicated that type II NKT cells and classical Treg cells were equally essential in suppressing antitumor responses (117).

The different cytokine profiles between type I and type II iNKT cells were better characterized in a murine model of B-cell lymphoma, where type I iNKT secreted primarily IFN-γ, and type II iNKT cells secreted TGF-β and IL-13 (78). Furthermore, a balance between the two subsets allowed for adequate tumor immunosurveillance, as demonstrated by the enhanced mortality of tumor-bearing mice that are deficient in type I NKT cells, but retain type II NKT cells (78).

In exploring αGC-induced anergy of iNKT cells, a distinct subset of regulatory iNKT cells was identified, now termed NKT10 cells (118). As the name suggests, this population of NKT cells secretes IL-10 upon antigenic stimulation and is able to increase the tumor burden in mice challenged with B16 melanoma cells (118). In line with these results, a recent report noted that the absence of iNKT cells correlated with reduced number of intestinal polyps in a murine model of colorectal cancer (119). It was shown that IL-10 producing iNKT cells—reminiscent of the NKT10 cell subset—were enriched within polyps (119). Furthermore, these cells lack the NKT cell transcription factor PLZF, in keeping with recent findings that the PLZF is absent in regulatory iNKT cells in adipose tissue, where they secrete IL-2 and IL-10, control the number of classical Tregs, and promote M2 polarization of adipose-resident macrophages (120).

## Manipulating iNKT Cells in Cancer Immunotherapy

Given their essential role in antitumor responses, iNKT cells are suitable targets for cancer immunotherapy research. Most studies have mainly focused on the adjuvant behavior of iNKT cells, in particular efficient methods of αGC delivery, often in combination with tumor antigens, to trigger an all-encompassing immune response against the tumor. However, more recent studies have focused on harnessing iNKT cells in new, promising cancer immunotherapies.

Although αGC is a naturally occurring iNKT cell agonist, which enhances the adjuvant effect of iNKT cells in cancer, there is a focus on identifying stronger iNKT cell agonists, either by modifying αGC or identifying novel molecules based on medicinal chemistry programs. Such efforts are supported by the structural knowledge of CD1d bound to αGC and of the iNKT-TCR either in isolation or during cognate interaction with CD1d–lipid complexes (29, 121–123). In screening a panel of αGC analogs for enhanced iNKT cell activation, several iNKT cell agonists were characterized that produce a strong Th1 response from iNKT cells. One of such compounds features an aromatic ring (or, more specifically, a phenyl ring) within the acyl tail (124–127) and is currently entering clinical trials as a vaccine adjuvant (128).

Since the polar head group of CD1d-bound lipids is key for recognition by the iNKT-TCR, cellular enzymes that might either catabolize some iNKT-cell agonists or redirect them away from lipid–antigen presentation pathways might in part drive suboptimal iNKT cell responses. This observation has fueled work that led to the identification of a novel class of iNKT cell agonists that possess non-carbohydrate structures coupled to the ceramide moiety (129). One of these compounds, threitol-ceramide (ThrCer), which was shown to be very efficient in augmenting antigen-specific T cell responses and minimizing iNKT cell overstimulation and iNKT cell-dependent DC lysis, is capable of rectifying the deficiencies of αGC (130). Recent results have shown that incorporating the head group of ThrCer into a conformationally more restricted six-membered ring results in significantly more potent non-glycosidic analogs. In particular, Thr-6-Cer (IMM60) was found to promote strong antitumor responses and to induce a more prolonged stimulation of iNKT cells than does the canonical αGC, achieving an enhanced T-cell response at lower concentrations compared with αGC both *in vitro*, using human iNKT cell lines, and *in vivo*, using C57BL/6 mice (35). The synthetic non-glycolipid IMM60 is currently entering clinical trials in melanoma and non-small-cell lung cancer patients in combination with anti-PD1 blocking antibodies. In addition, given that the coupling of iNKT cell agonists with PLGA nanoparticles enhances their immune adjuvant potential by orders of magnitude (131), a phase I clinical trial in ovarian cancer and prostate cancer patients will be carried out with IMM60 conjugated to PLGA nanoparticles with full length NY-ESO-1 protein.

Efficient delivery of potent lipid iNKT cell agonists is essential in manipulating the adjuvant effects of iNKT cells. Optimizing delivery methods and combining the stimulatory lipid with tumor-specific antigens are critical to ensure that the adjuvanted immune response is targeted predominantly toward the tumor. In that vein, the use of exosomes as a means of codelivering αGC and ovalbumin has proved highly successful in reducing the tumor burden and increasing survival in mice inoculated with OVA-expressing melanoma compared with injection of soluble αGC and OVA together (132). Since exosomes naturally bear surface markers to direct them to a particular destination—and for this reason are utilized by breast cancer cells themselves to create a “metastatic niche” at a location of future metastasis (133)—they make excellent conduits for delivery of this potential “cancer vaccine” directly to the tumor site, while perhaps protecting the contents from degradation during delivery. Synthetic nanoparticle delivery systems also hold great promise. In a construct similar to exosomes, delivery of αGC or TLR 3 and 7/8 agonists polyI:C and R848, and OVA in biodegradable poly(lactic-co-glycolic acid) nanoparticles proved efficient in stimulating CD8^+^ antigen-specific T cell responses against OVA-B16 independent of CD4^+^ T cell help (131). Encapsulation of the contents was essential, as injection of a mixture of αGC, TLR ligands, and OVA did not induce a comparable antitumor T cell response (131).

As previously mentioned, a major pitfall in using glycolipid antigens, specifically αGC, as adjuvants in cancer immunotherapy is the induction of iNKT cell anergy, as defined by reduced IFN-γ secretion upon secondary exposure. The delivery of αGC, typically as an injection of free lipid particles that might be taken up and presented by a variety of antigen-presenting cells, might contribute to this issue. An alternate delivery method would involve the intravenous injection of autologous DCs preloaded with αGC. In a clinical study involving five late-stage cancer patients, injection of αGC-loaded DCs led to the robust proliferation of iNKT cells, sustained IFN-γ secretion, and enhanced antigen-specific CD8^+^ T-cell expansion *ex vivo*, as compared to injection of unpulsed DCs (134, 135). It is suggested that the extent of anergy induction is dependent on the type of antigen-presenting cells that present αGC, with B cells reportedly inducing a higher degree of anergy than DCs (135, 136). In addition to the augmented Th1 response upon injection of αGC-loaded DCs (137), perhaps upon secondary stimulation with αGC-pulsed DCs, anergic responses would be reduced. Alternatively, targeting iNKT cell agonists to DCs through nanoparticle formulations has been shown to overcome iNKT cell anergy (138).

The recent emergence of monoclonal antibody therapies to checkpoint regulators has revolutionized the field of cancer immunotherapy, particularly antibodies targeting the PD1–PD1L axis and CTLA4. While these therapies are studied predominantly in the context of CD8^+^ cytotoxic T cells, iNKT cells are not exempt from their influence. Much like conventional T cells, iNKT cells upregulate PD1 on their cell surface upon activation as means of eventually resolving the immune response (139). Blockade of PD1 using anti-PD1 antibodies injected simultaneously with αGC results in iNKT cell activation and prevents iNKT cell anergy, a common occurrence after potent αGC stimulation (139–141). In fact, blockade of PD1 during αGC-mediated iNKT cell activation in a B16 melanoma mouse model leads to a persistent antimetastatic immune response (139).

Another emerging T cell-based cancer immunotherapy centers on the chimeric antigen receptor (CAR) T cell therapy. CAR T cell therapy works on the principle that genetically engineered CD8^+^ T cells expressing TCRs specific for a tumor antigen fused to their native CD3 domain or modified with the endodomain of a costimulatory molecule can become activated and expand into a population of tumor-specific CD8^+^ cytotoxic T cells (142). This approach has recently been applied to iNKT cells (143). A CAR specific for GD2 ganglioside, an abundant neuroblastoma antigen, was expressed in primary human iNKT cells (143). CAR.GD2 iNKT cells took on a Th1 profile and localized directly in the tumor site when transplanted in NSG mice (143). CAR.GD2 iNKT cells were highly cytotoxic against neuroblastoma cells, and when fused with CD28 and 41BB endodomains, they increased long-term survival in a murine model of the disease (143). With conventional T cells, a frequent adverse effect of CAR therapy in NSG mice is that adoptive transfer of the engineered T cells can induce graft-versus-host disease (GVHD) (143). However, there is no evidence of GVHD in *in vivo* models utilizing CAR.GD2 iNKT cells (143). For this reason, CAR iNKT cells might become an alternative to conventional T cells as vectors for CAR therapy. So far, however, CAR iNKT cell therapy has not been translated into clinical trials due to a poor understanding of the mechanisms underlying their *in vivo* proliferation and persistence (144). There have been no clear markers to differentiate effector and memory iNKT cells (144). Recently, a subset of iNKT cells that express the adhesion marker CD62L (also found in naïve and central memory T cells) has been identified (144). As expected, this population rapidly expands and can persist upon stimulation (144). In iNKT cells transduced to express a CD19.CAR, it was the CD62L^+^ population that achieved persistent activation and proliferation *in vivo* and was responsible for lymphoma and neuroblastoma regression (144).

## Conclusion

Recent results have indicated that therapies harnessing iNKT cells seem generally well tolerated by mice and humans. There are still many unanswered questions in the field of iNKT cell therapies that demand full investigation, such as the optimal route of administration, formulation of dosing intervals, etc. Although preclinical studies in animal models may help answer these questions, ultimately, appropriately designed clinical trials in humans will guide protocol optimization. Our ability to manipulate these cells in antitumor therapeutics is critically dependent on our understanding of iNKT cell biology, including the factors that activate and regulate these cells during sterile and non-sterile conditions; the strong immunomodulatory ability of iNKT cells begs the question as to whether their activation in cancer patients, in combination with immune check point inhibitors, can enhance the frequency and quality of neo-antigen tumor-specific CD8^+^ and CD4^+^ T cell responses. The identification, optimization, formulation, and clinical use of iNKT cell agonists that promote Th1 immune responses should be a high priority in future clinical trials.

## Author Contributions

MB wrote the manuscript, while MS and VC contributed to the writing and editing of the text.

## Conflict of Interest Statement

The authors declare that the research was conducted in the absence of any commercial or financial relationships that could be construed as a potential conflict of interest.
